# A pilot and exploratory study on the effects of a board-game intervention on early mathematical reasoning in preschool children

**DOI:** 10.3389/fpsyg.2026.1862028

**Published:** 2026-06-12

**Authors:** Hacer Koç, Ensar Yıldız, Sibel Karabekmez

**Affiliations:** 1 Institute of Educational Sciences, Sivas Cumhuriyet University, Sivas, Türkiye; 2 Faculty of Education, Sivas Cumhuriyet University, Sivas, Türkiye; 3 Department of Child Care and Youth Services, Agri Ibrahim Cecen University, Agri, Türkiye

**Keywords:** board games, early mathematics, mathematical reasoning, preschool, reasoning skills

## Abstract

**Introduction:**

This study examined the effectiveness of a structured board-game intervention on early mathematical reasoning skills in preschool children.

**Methods:**

This pilot and exploratory study used a quasi-experimental pretest-posttest control group design, 24 children aged 60–71 months were assigned to experimental (*n* = 12) and control (*n* = 12) groups. The intervention, implemented over 6 weeks, included five board games targeting cognitive and reasoning processes. Data were collected using the Early Mathematical Reasoning Skills Assessment Tool and analyzed using ANCOVA, change score comparisons, and interaction-based regression with Johnson-Neyman techniques.

**Results:**

Controlling for pretest scores obtained from the Early Mathematical Reasoning Skills Assessment Tool, the experimental group showed significantly higher posttest mathematical reasoning performance than the control group. Moderation analyses indicated stronger effects for children with medium and high initial skill levels. Subdimension analyses revealed larger gains in data analysis-probability than in measurement.

**Discussion:**

Overall, the findings suggest that board-game activities can effectively support early mathematical reasoning, although additional support may be needed for measurement-related skills. However, the findings should be interpreted as preliminary due to the pilot and exploratory nature of the study and require validation through larger-scale research.

## 1 Introduction

Early childhood is a critical period during which the foundations of children’s mathematical thinking and reasoning skills are established. Number knowledge, pat-tern awareness, and quantity comprehension acquired at this stage provide a strong basis for more advanced mathematical learning. For this reason, effective interventions implemented early on can contribute to long-term academic gains (Nunes et al., 2009).

Play-based learning is widely recognized as both a natural and effective instructional approach in early childhood education. Structured play activities such as board games allow children to explore mathematical processes including number recognition, using ordinal knowledge, estimating and comparing, and developing strategies. The repetitive and socially interactive nature of these games enables mathematical concepts to be reinforced experientially (Skwarchuk et al., 2014).

From a theoretical standpoint, the effectiveness of play-based learning can be explained through several core mechanisms. Vygotsky’s (1978) sociocultural theory emphasizes that learning occurs through social interaction and through the support pro-vided by more knowledgeable others (ZPD). In this sense, board games enable children to acquire reasoning strategies through joint problem-solving and linguistic scaffolding offered by peers and adults. On the other hand, Piaget’s perspective suggests that “rule-based games” support the development of cognitive structures and forms of logical thinking (Piaget, 1962). Thus, board games activate both social and cognitive developmental pathways simultaneously. Taken together, these two theoretical frameworks provide an explanatory foundation for how and why games can support mathematical reasoning.

### 1.1 Mathematical reasoning skills in early childhood

Reasoning is viewed as a mental process through which an individual attempts to make inferences about a particular concept by using available information (Başara Baydilek, 2016). During the reasoning process, the individual shapes information with-in the framework of their own logic and connects the process to a conclusion by establishing cause-effect relationships (Ergül and Artan, 2015).

Mathematical reasoning in early childhood encompasses the thinking processes children use to make sense of problems involving number, quantity, pattern, space, and relationships. These processes involve not only performing calculations but also under-standing concepts, forming connections, and making generalizations (National Research Council, 2009). Individual differences that emerge early on meaningfully predict later school success. Mathematics skills at school entry predict subsequent academic achievement more strongly than other readiness components such as reading or attention (Duncan et al., 2007). Therefore, supporting mathematical reasoning in early childhood is considered critical for both individual development and equity/achievement (National Research Council, 2009; Sarama and Clements, 2009).

### 1.2 The relationship between board games and mathematical reasoning skills

Rapid global changes have heightened the need to raise individuals who can adapt, think critically, collaborate, and reason effectively. Mind and intelligence games are important instructional tools supporting these skills (Kul and Kel, 2021), and introducing them early contributes significantly to cognitive development (Gülle and Vatansever Bayraktar, 2023). Board games, a major category within this domain, provide skill development opportunities across ages (Treher, 2011) and can be effectively integrated into classroom instruction when aligned with learning goals (Wong and Yunus, 2021).

Play-based approaches are grounded in Vygotskian perspectives, where structured interactions and scaffolding foster higher-order reasoning (Bodrova and Leong, 2018). Experimental research has shown that linear number board games enhance early numerical competencies such as number-line estimation, magnitude comparison, and counting (Ramani and Siegler, 2008). Systematic reviews similarly report generally positive effects of board games on mathematics learning, though impacts vary by game type, implementation frequency, and instructional context (Balladares et al., 2024; DePascale and Ramani, 2025).

Board games particularly support reasoning domains involving patterning, grouping, sequencing, graph interpretation, and probability, as young children can make basic probabilistic judgments (Nikiforidou and Pange, 2009). In contrast, measurement concepts require concrete physical interaction, and because most games lack such experiences, gains in this area tend to be weaker (Ergül and Artan, 2015).

Developmentally informed mathematical instruction emphasizes tailoring support to children’s current reasoning levels (Sarama and Clements, 2009). Well-guided activities can promote transfer of strategies, helping children generalize learned relation-ships across contexts rather than relying on rote procedures (Baroody et al., 2016).

### 1.3 Current study

Preschool children’s mathematical reasoning has been investigated extensively in Türkiye and internationally. In the Turkish context, studies have examined domains such as measurement and data analysis-probability (Ergül and Artan, 2015), compared children with special needs and typically developing peers (Piştav Akmeşe et al., 2020a, 2020b), and explored attention (Gözüm, 2017), executive functions (Özgür, 2023), and patterning skills (Yayla, 2022) as predictors of reasoning.

Research on play-based approaches has also highlighted their role in supporting children’s learning, including problem solving (Güngör and Tuğrul, 2020) and early literacy (Şen, 2020). However, studies directly evaluating structured board-game interventions targeting mathematical reasoning in early childhood remain scarce. International findings provide strong evidence that play-based learning fosters numerical development. Linear number-board game studies demonstrated substantial, replicable gains in number-line estimation and numerical knowledge (Ramani and Siegler, 2008; Laski and Siegler, 2014). Broader work emphasizes the cognitive, social, and emotional value of play in early education, positioning it as a purposeful pedagogical tool (Ginsburg, 2007; Hirsh-Pasek et al., 2009). Despite the generally positive effects of such interventions, heterogeneity in game type, dosage, design, and assessment complicates strong conclusions about optimal implementation. Highlighting their multidomain contributions, O'Neill and Holmes (2022) noted developmental benefits of tabletop games while underscoring that evidence in this area remains emergent. Moreover, board games are still rarely used in preschool classrooms, indicating a need for scientifically evaluated, educationally designed games (Balladares et al., 2024).

Methodologically, most early-childhood intervention studies focus on overall group differences without considering variability in effects across initial ability levels. The Johnson-Neyman (J-N) technique addresses this limitation by identifying the specific score ranges in which an effect becomes statistically significant (Carden et al., 2017; Montoya, 2019). Yet, early-childhood studies employing J-N remain limited (Brock and Kochanska, 2016; Chang and Jahng, 2014; Pan et al., 2025), typically focusing on socioemotional and behavioral constructs. Therefore, applying this approach to a play-based mathematics intervention represents a novel and original contribution. In addition, considering the practical and contextual constraints of conducting controlled interventions in early childhood settings, the present study was designed as a pilot and exploratory investigation. The aim is to provide preliminary evidence regarding the effectiveness of structured board-game interventions and to identify potential patterns that can inform future large-scale studies. Within this frame-work, the study seeks to answer the main research question, “What is the effect of board game activities on the mathematical reasoning skills of children aged 60–71 months?” along with the following sub-questions.

- What is the effect of board-game activities on the early mathematical reasoning skills of children aged 60–71 months?
- What is the effect of board-game activities on the early mathematical reasoning sub-dimensions (data analysis-probability and measurement) of children aged 60–71 months?
- At which initial skill levels do the effect of board-game activities on the mathematical reasoning abilities of children aged 60–71 months emerge more prominently?

## 2 Methods

This study employed a pretest-posttest control group quasi-experimental design to examine the effects of board-game activities on children’s early mathematical reasoning skills. In such designs, experimental and control groups are created based on similar characteristics rather than random assignment, distinguishing them from true experiments (Fraenkel and Wallen, 2006).

### 2.1 Participants

The participants consisted of 24 typically developing children aged 60–71 months who were selected through convenience sampling and were enrolled in kindergarten classes within a primary school affiliated with the Ministry of National Education in a province located in the Central Anatolia Region of Türkiye (n_experimental_ = 12 [Girls: 5, Boys: 7]; n_control_ = 12 [Girls: 5, Boys: 7]). The school was selected based on accessibility and feasibility criteria. Within the selected school, two preschool classrooms were included in the study. Given the pilot and exploratory nature of the study, the sample size was determined based on accessibility and feasibility considerations. The primary purpose was not to produce generalizable conclusions, but to examine initial effectiveness, feasibility, and potential patterns of impact to inform future large-scale studies.

### 2.2 Data collection tool

#### 2.2.1 Early mathematical reasoning skills assessment tool

This tool, developed by Ergül and Artan (2015) to assess mathematical reasoning skills in children aged 60–74 months, includes 40 items: 21 in the measurement subscale (length, weight, area, volume, and time concepts) and 19 in the data analysis-probability subscale (shape discrimination, graph construction, picture prediction, graph reading, and probability concepts). It is administered individually, with responses scored be-tween 0 and 5. Reported psychometric properties indicate strong reliability (test–retest = 0.98; Cronbach’s *α* = 0.85; inter-rater *κ* = 0.85). In the present study, pretest scores yielded Cronbach’s α = 0.948, indicating high internal consistency (George and Mallery, 2003).

### 2.3 Data collection

After receiving ethics committee approval, permissions were obtained from the Provincial Directorate of National Education. Following institutional approval, the selected school was contacted and administrative arrangements were completed in coordination with school staff. After obtaining parental consent, children who voluntarily agreed to participate were included in the study, and their assent was obtained. The implementation process was conducted in accordance with established ethical guide-lines for research with children (Flewitt, 2005; Shaw et al., 2011). Children were informed about the purpose of the study in an age-appropriate manner, and their voluntary assent was obtained prior to participation. The implementation was initiated at times when children were willing to participate, and they were informed that they could pause or withdraw from the activities at any time. During the activities, a supportive and non-hierarchical interaction was maintained by positioning at the same physical level as the child. To ensure confidentiality, all participants were anonymized using coded identifiers (e.g., C1, C2). Pretest data were administered individually in the school guidance room under counselor supervision using the Early Mathematical Reasoning Skills Assessment Tool (Ergül and Artan, 2015).

Prior to group assignment, pretest assessments were administered to children in both classrooms, and the results indicated no statistically significant differences between the classes at baseline. Based on the equivalence of pretest scores and to ensure feasibility and ecological validity, one classroom was assigned to the experimental condition and the other to the control condition. Because intact classrooms were used, potential teacher/classroom effects cannot be fully ruled out; therefore, findings should be interpreted with caution.

The experimental group received a six-week intervention (April–May 2025), 5 days a week for approximately 45 min per day, using the selected five games (Rondo Vario, Speed Cups, Detective Junior, Q-bitz Junior, Mangala). Posttests were conducted in the same setting and with the same procedures.

To ensure the intervention in the experimental group was carried out as planned and consistently, all applications were conducted according to a predetermined standard application plan. The same board games were used in the experimental classroom throughout the intervention, the activities were applied for the same duration and frequency, and the implementation process was regularly recorded. These application records were used to monitor the intervention’s adherence to the planned content and framework. The routine preschool education program was continued in the control group classroom. This arrangement aimed to prevent the mixing of intervention components between the groups. The implementation was conducted in a standardized manner to ensure consistency across sessions and to minimize potential implementation related variability.

Following the completion of the study, and in line with ethical considerations commonly adopted in experimental research, an additional six-week implementation period was conducted during which the intervention, having been demonstrated to be effective, was subsequently provided to the control group.

### 2.4 Procedure

The intervention implemented in the study was designed by selecting board games that were suitable for the developmental level of children aged 60–71 months and aligned with mathematical reasoning objectives. In this context, 10 commercially available board games designed for the preschool period (EQuilibrio, Rondo Vario, Mangala, Skillful Fingers, Detective Junior, Q-bitz Junior, Choo Choo Train, Candy, Block Buddies, and Speed Cups) were identified based on their appropriateness for the children’s age level and the cognitive skills targeted by the research. Expert opinions were obtained from two early childhood education specialists and one mind-and-intelligence-games specialist. Based on expert evaluations, the five board games judged to be most suitable for the targeted developmental skills were selected. These games, in the order in which they were played, are listed below:

#### 2.4.1 Rondo Vario

**Figure 1:**
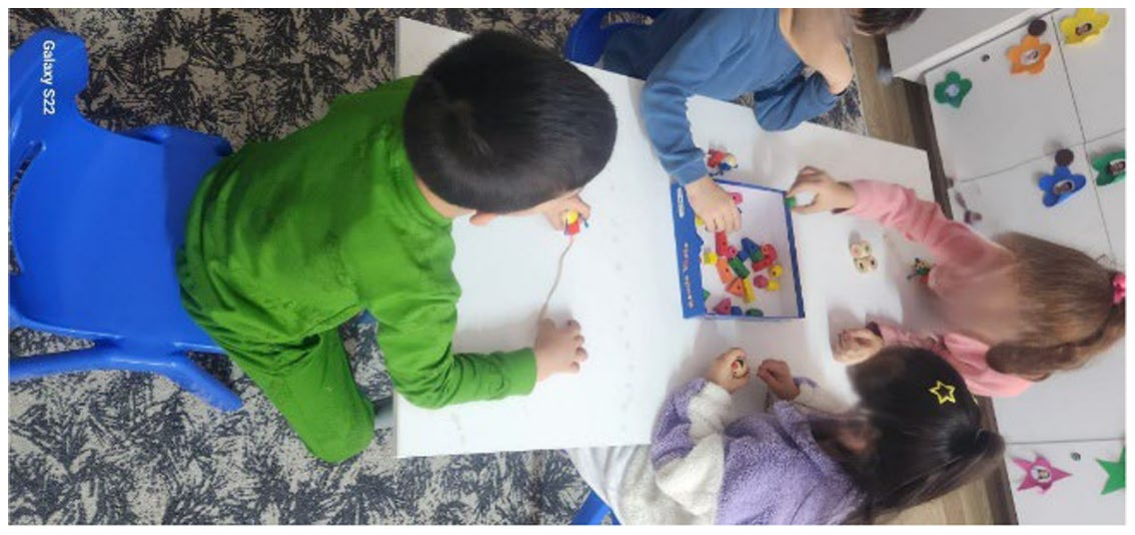
Rondo Vario game.

Rondo Vario is a matching game that fosters classification and pattern formation by color, shape, and size. As shown in Figure 1, players sequence objects using visual cues and establish logical relations; these demands promote inductive reasoning and visual attention.

#### 2.4.2 Speed Cups

**Figure 2:**
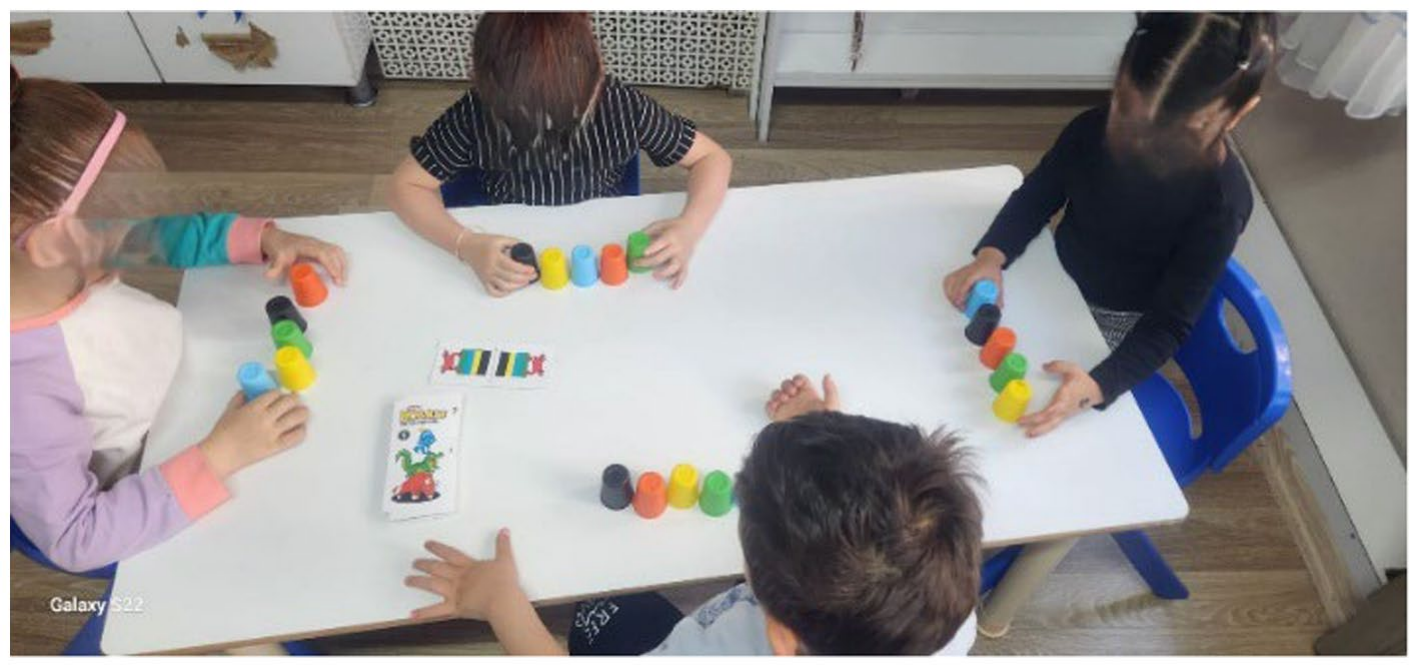
Speed Cups game.

It is a hand-eye coordination and speed game that requires children to recreate the color and pattern arrangement shown on a given card using cups as quickly as possible. It supports visual perception, pattern recognition, sequencing, and processing speed. It also strengthens attention control and the ability to respond in accordance with rules (Figure 2).

#### 2.4.3 Detective Junior

**Figure 3:**
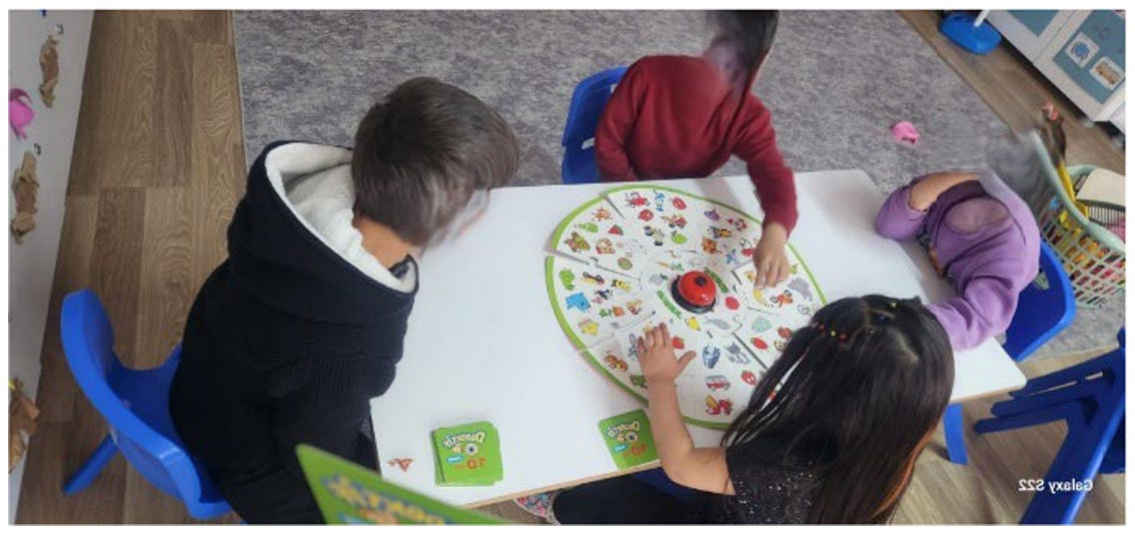
Detective Junior game.

It is an inference game based on analyzing visual clues to identify the correct object or person. Players try to reach the correct answer by using logical reasoning and probability-evaluation processes. With these features, Detective Junior is designed to develop children’s deductive reasoning and problem-solving skills (Figure 3).

#### 2.4.4 Q-bitz Junior

**Figure 4:**
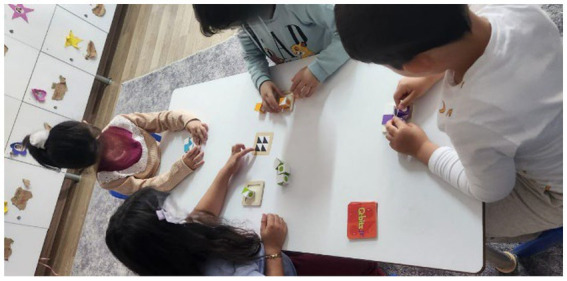
Q-bitz Junior game.

It is a game that simultaneously supports spatial reasoning, visual patterning, attention, and motor coordination skills. Children are expected to recreate a given visual pattern using cubes with shapes on them. The game supports children’s ability to develop strategies and explore multiple solution paths (Figure 4).

#### 2.4.5 Mangala

**Figure 5:**
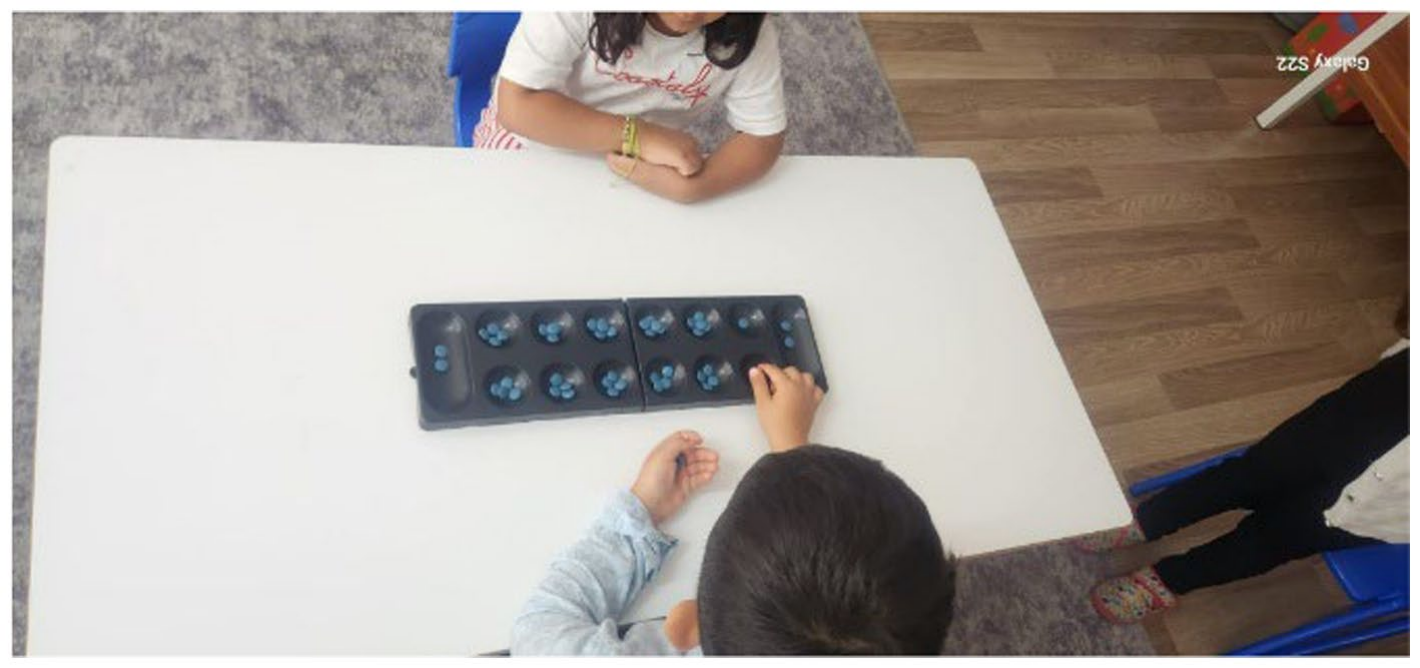
Mangala game.

Mangala is a traditional, strategy-based intelligence game that has been played in Turkish culture for centuries. Played between two players, the game is based on moving stones in the small pits in front of each player according to specific rules, with the goal of collecting the highest number of stones. During the game, children actively engage counting, making predictions, planning, sustaining attention, and developing strategic thinking skills (Figure 5).

During the intervention, each board game was implemented for 1 week across five consecutive sessions. In the first session of each week, the game was introduced, the rules were explained, and an example round was played to ensure comprehension. On subsequent days, the children continued in three groups of four, with one game set per group; however, during the Mangala week, each group was divided into two, forming six groups of two due to its two-player structure. This organization enabled simultaneous participation, promoted cooperation, and ensured active engagement. Gameplay was monitored, guidance was provided, and children’s strategies and interactions were observed. In the sixth week, a rotation format was implemented. Five game stations were set up, and three groups of four children rotated approximately every 8–10 min within the 45-min session. At the Mangala station, children played in pairs due to its two-player structure. This format enabled all children to revisit each game while maintaining engagement. Both the experimental and control groups followed the rou-tine preschool education program used in all kindergartens nationwide throughout the study period. In addition to this usual educational process, the experimental group par-ticipated in board game-based activities during planned sessions and durations. The control group received no additional interventions outside of the routine educational process.

**Figure 6:**
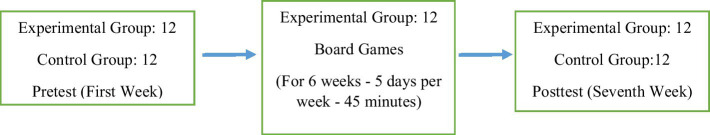
Data collection procedure.

Observations indicated consistently high engagement in the experimental group. Children adapted quickly to games such as Rondo Vario and Speed Cups, while Mangala initially required more guidance due to its strategic complexity; however, by the end of the week, all children could participate successfully. These observations suggest that repeated exposure and peer interaction facilitated learning and motivation (Figure 6).

### 2.5 Data analysis

As part of the data analysis, normality was tested using the Shapiro–Wilk test, recommended for small-to-medium samples (Shapiro and Wilk, 1965). Homogeneity of variances was examined with Levene’s test. Following these assumption checks, descriptive statistics, an independent samples t-test, and ANCOVA were conducted. To assess the group × pretest interaction, a multiple regression model was estimated, and the Johnson-Neyman technique was applied. Additionally, change-score analysis (*Δ* = posttest-pretest) was performed as an assumption-free complementary approach. Analyses were conducted in SPSS 26 IBM and R 4.5.1 (R Core Team, 2025). For ANCOVA, the car package (Fox and Weisberg, 2019) was used; for effect sizes, effect size (Ben-Shachar et al., 2020); for interaction and Johnson-Neyman analyses, interactions (Long, 2024a) and jtools (Long, 2024b); for data import haven (Wickham et al., 2025); and for visualizations, ggplot2 (Wickham, 2016). A *post hoc* power analysis was performed using G*Power 3.1 (Faul et al., 2009). To report effect sizes comprehensively, Cohen’s d, Hedges’ g, Glass’s Δ, ω^2^, and η^2^_p_ values were obtained via the GAMLj module in Jamovi.

### 2.6 Ethical considerations

This study strictly adhered to ethical standards throughout the entire research process. Participation of children was based on voluntary consent. In addition, written informed consent was obtained from parents for their children’s participation. No identifying information about participants was recorded at any stage, and all data were anonymized and securely stored in a protected digital environment.

## 3 Results

The study investigated the effect of board-game activities on children’s early mathematical reasoning skills as measured by the Early Mathematical Reasoning Skills Assessment Tool. The results are presented through comparisons of pretest and posttest scores obtained from the total scale and its subscales.

As shown in Table 1, an independent samples t-test revealed no significant difference between the experimental (*M* = 90, SD = 22.41) and control (*M* = 103.08, SD = 19.59) groups in pretest Early Mathematical Reasoning Skills Assessment Tool total scores, *t*(22) = 1.52, *p* = 0.142, 95% CI [−30.90, 4.74]. Although the effect size was moderate (Hedges’ *g* = −0.60), the confidence interval included zero, indicating non-significance.

**Table 1:** Pretest scores and independent samples t-test results by group with effect sizes.

Subscale	Group	*N*	*M*	SD	*t*	*df*	*p*	Hedges’ *g* [95% CI]
Total	Experimental	12	90	22.413	1.523	22	0.142	−0.60 [−1.39, 0.20]
	Control	12	103.08	19.589				
Measurement	Experimental	12	57.08	12.75	1.259	22	0.221	−0.50 [−1.28, 0.30]
	Control	12	63.00	10.12				
Data analysis-probability	Experimental	12	32.92	10.23	1.752	22	0.094	−0.69 [−1.48, 0.12]
	Control	12	40.08	9.80				

No significant difference was identified between the groups in terms of pre-test total and subscale scores (*p* > 0.05; Levene’s Test: *F*(1, 22) = 0.65, *p* = 0.429). However, the control group had higher means in all subscales, and the Hedges’ *g* values ranged from small to medium.

Before comparing posttest scores obtained from the Early Mathematical Reasoning Skills Assessment Tool using ANCOVA, assumption checks were per-formed. Pretest-posttest scores met normality based on the Shapiro–Wilk test, and a strong positive correlation was found between them (*r* = 0.89, *p* < 0.001). Levene’s test confirmed homogeneity of variances (*F*(1, 22) = 0.85, *p* = 0.366). Accordingly, ANCOVA was conducted while controlling for pretest scores. A *post hoc* power analysis using G*Power 3.1 (Faul et al., 2009) showed high power (1–*β* ≈ 0.99) based on the observed effect size (partial η^2^ = 0.586; *f* = 1.19, *α* = 0.05, *N* = 24), demonstrating adequate sensitivity to detect group differences.

ANCOVA results showed that pretest scores significantly predicted posttest performance, *F*(1, 21) = 93.44, *p* < 0.001, η^2^_p_ = 0.816, ω^2^ = 0.801. After controlling for the covariate, the group effect was also significant, *F*(1, 21) = 29.75, *p* < 0.001, η^2^_p_ = 0.586, ω^2^ = 0.556. The observed power was 0.99 and was confirmed with a post hoc G*Power analysis (Cohen’s f = 1.19, 1–*β* = 0.999), indicating a large intervention effect, explaining 59% of the variance in posttest scores. For the measurement subscale, pretest scores significantly predicted posttest scores, *F*(1, 21) = 142.31, *p* < 0.001, η^2^_p_ = 0.871, ω^2^ = 0.860, while the group effect remained significant at a small-to-medium level, *F*(1, 21) = 17.39, *p* < 0.001, η^2^_p_ = 0.453, ω^2^ = 0.416. In the data analysis–probability subscale, significant effects were again observed for both pretest, *F*(1, 21) = 44.37, *p* < 0.001, η^2^_p_ = 0.682, ω^2^ = 0.656, and group, *F*(1, 21) = 27.70, *p* < 0.001, η^2^_p_ = 0.569, ω^2^ = 0.537, reflecting a medium-to-large intervention effect.

Adjusted posttest means indicated that the experimental group achieved significantly higher total scores than the control group. The adjusted mean total score of the experimental group (M_adj = 149.37, SE = 3.45, 95% CI [142.20, 156.53]) was significantly higher than that of the control group (M_adj = 122.13, SE = 3.45, 95% CI [114.97, 129.30]), *t*(21) = 5.45, *p* < 0.001, with a mean difference of 27.23 points. The effect size was large (η^2^_p_ = 0.586, ω^2^ = 0.556), indicating a strong overall intervention effect.

For the measurement subscale, the experimental group (M_adj = 81.50, SE = 1.40, 95% CI [78.60, 84.40]) also outperformed the control group (M_adj = 73.10, SE = 1.40, 95% CI [70.20, 76.00]), *t*(21) = 4.17, *p* = 0.0004, yielding an 8.40-point mean difference and a small-to-medium effect size (η^2^_p_ = 0.453, ω^2^ = 0.416).

Similarly, in the data analysis-probability subscale, adjusted mean scores favored the experimental group (M_adj = 67.60, SE = 2.39, 95% CI [62.60, 72.60]) also outperformed the control group (M_adj = 49.20, SE = 2.39, 95% CI [44.30, 54.20]), *t*(21) = 5.26, *p* < 0.0001, reflecting an 18.30-point mean difference and a medium-to-large effect (η^2^_p_ = 0.569, ω^2^ = 0.537).

To examine the homogeneity of regression slopes assumption, the interaction be-tween group and pretest scores was tested. For the total score, the group × pretest interaction was statistically significant, *F*(1, 21) = 6.66, *p* = 0.022, with a significant slope difference, *t*(21) = 2.48, *p* = 0.022. The pretest–posttest slope was steeper in the experimental group (*b* = 1.02, *p* < 0.001) than in the control group (*b* = 0.58, *p* < 0.001), indicating a violation of the homogeneity of regression slopes assumption and suggesting that the intervention effect varied by initial skill level.

For the measurement subscale, the interaction was not statistically significant, *F*(1, 21) = 3.15, *p* = 0.091; *t*(21) = 1.78, *p* = 0.091. Although pretest–posttest relationships were significant in both groups (experimental: *b* = 0.84, *p* < 0.001; control: *b* = 0.56, *p* < 0.001), the slopes did not significantly differ, indicating that the homogeneity assumption was met.

In contrast, for the data analysis-probability subscale, the group × pretest interaction was significant, *F*(1, 21) = 5.63, *p* = 0.028, *t*(21) = 2.37, *p* = 0.028, with a steeper slope in the experimental group (*b* = 1.22, *p* < 0.001) than in the control group (*b* = 0.58, *p* < 0.001). This finding indicates that the intervention effect varied depending on the pretest level.

Overall, significant differences in regression slopes were observed for the total score and the data analysis-probability subscale, whereas no significant slope difference emerged for the measurement subscale, suggesting that the intervention exerted differential effects across levels of initial performance.

Table 2 shows that the interaction regression model predicting posttest Early Mathematical Reasoning Skills Assessment Tool scores is significant. Pretest scores strongly predict posttest performance (*b* = 1.11, *β* = 0.89, *t* = 10.35, *p* < 0.001, 95% CI [0.89, 1.33]), indicating that the pretest level is the primary determinant of posttest achievement. Although the main effect of group is non-significant (*b* = −12.13, *β* = −0.25, *t* = −1.14, *p* = 0.267), the significant group × pretest interaction (*b* = 0.26, *β* = 0.43, *t* = 2.48, *p* = 0.022, η^2^_p_ = 0.235, ω^2^ = 0.034) shows that the intervention effect differs based on initial skill level, with greater gains observed among children who started at higher pretest levels.

**Table 2:** Interaction regression analysis results for predicting posttest scores.

Outcome variable	Predictor	*B*	SE B	*β*	*t*	*p*	95% CI	η^2^*_p_*	ω^2^
Total	Intercept	30.94	10.62	–	2.91	0.008	[8.36, 53.52]		
	Group (control vs. experimental)	−12.13	10.62	−0.25	−1.14	0.267	[−34.35, 10.09]	0.296	0.049
	Pretest	1.11	0.11	0.89	10.35	< 0.001	[0.89, 1.33]		
	Group × pretest	0.26	0.11	0.43	2.48	0.022	[0.04, 0.48]	0.235	0.034
Data analysis-probability	Intercept	18.65	5.87	–	3.18	0.004	[6.40, 30.90]		
	Group (control vs. experimental)	−4.24	5.87	−0.10	−0.72	0.478	[−16.69, 8.21]	0.372	0.116
	Pretest	1.13	0.15	0.82	7.29	< 0.001	[0.82, 1.44]		
	Group × pretest	0.37	0.15	0.46	2.37	0.028	[0.04, 0.70]	0.220	0.050

Model summary: *R*^2^ = 0.87, Adj. *R*^2^ = 0.85, *F*(3, 20) = 43.65, *p* < 0.001. Dependent variable: Posttest (total). Model summary: *R*^2^ = 0.78, Adj. *R*^2^ = 0.75, *F*(3, 20) = 24.11, *p* < 0.001. Dependent variable: Posttest (data analysis-probability).

A similar pattern emerged for the data analysis–probability subscale. Pretest scores were a strong predictor of posttest performance (*b* = 1.13, *β* = 0.82, *t* = 7.29, *p* < 0.001, 95% CI [0.82, 1.44]), the group main effect was not significant (*b* = −4.24, *β* = −0.10, *t* = −0.72, *p* = 0.478), and the interaction was significant (*b* = 0.37, *β* = 0.46, *t* = 2.37, *p* = 0.028, η^2^_p_ = 0.220, ω^2^ = 0.050). These results similarly indicate that children with stronger initial competencies benefited more from the intervention.

Across both total scores and the data analysis–probability subscale, effect sizes suggest a small-to-moderate interaction effect.

The Johnson-Neyman technique revealed that the effect of group on posttest scores was significant when pretest values were at or above approximately 72.98–73, and non-significant below this threshold, as shown in Table 3. Simple Slopes analyses further indicated that at the 25th percentile of pretest (*b* = −18.07, SE = 5.81, *p* = 0.006, 95% CI [−30.15, −5.90]) and higher levels, the group effect was negative and statistically significant, whereas at the 10th percentile of pretest, the group effect was nonsignificant (*b* = −11.47, SE = 7.77, *p* = 0.155). These results suggest that the intervention effect was particularly pronounced among children with medium to high pretest scores.

**Table 3:** Johnson–Neyman technique and simple slopes results.

Pretest level (percentile)	*b* (group effect)	SE	*t*	*p*	95% CI [LL, UL]
10%	−11.47	7.77	−1.48	0.155	[−27.68, 4.74]
25%	−18.07	5.81	−3.10	0.006	[−30.15, −5.90]
50%	−29.64	4.54	−6.42	0.000	[−38.60, −19.66]
75%	−36.26	3.57	−6.29	0.000	[−48.30, −24.24]
90%	−37.43	6.07	−6.16	0.000	[−50.10, −24.76]

Pretest, baseline test score; Group effect, experimental vs. control group comparison.

**Figure 7:**
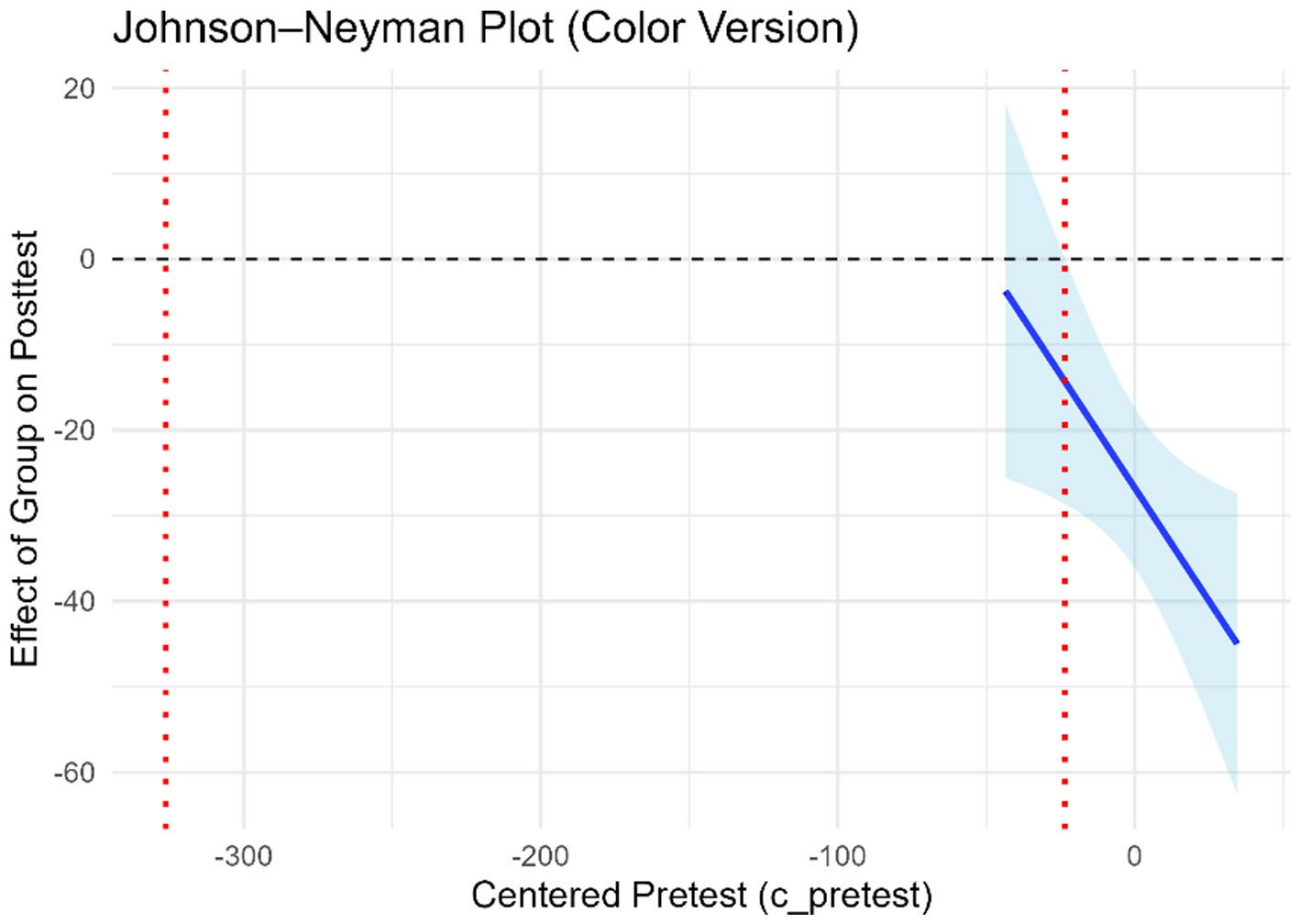
Johnson–Neyman plot. The Johnson–Neyman analysis yielded an interaction plot. In the graph, the blue line represents the effect on the dependent variable, and the light blue band shows the 95% confidence interval around that effect. The horizontal black dashed line indicates the reference point where the effect is zero. The vertical red dashed lines mark the threshold values that delimit the range within which the group effect is statistically significant.

The Johnson-Neyman analysis shows that the difference between the experimental and control groups becomes statistically significant at pretest scores of 72.98 and above (*p* < 0.05), as shown in Figure 7. In other words, group differences are not significant at low pretest levels, whereas significant advantages emerge for the experimental group at moderate and high pretest levels.

The change score analysis (*Δ* = posttest - pretest) revealed a significant main effect of group, *F*(1, 22) = 28.14, *p* < 0.001, R^2^ = 0.56, indicating that the experimental group demonstrated significantly greater improvement in reasoning skills compared to the control group. This finding further supports the robustness of the effects observed in the ANCOVA and Johnson-Neyman analyses.

## 4 Discussion

The results demonstrated that the board-game intervention led to a large and statistically significant improvement in children’s mathematical reasoning skills, supporting the effectiveness of play-based approaches in early childhood education (Alotaibi, 2024; Bodrova and Leong, 2018; Haoyue and Oyam, 2024; Sutoyo, 2017).

The findings of this study should be interpreted within the context of its pilot and exploratory design. Pilot studies are particularly valuable in early childhood research, where controlled experimental implementations are often constrained by practical and ethical considerations. In this respect, the present study provides preliminary but methodologically grounded evidence regarding the potential of structured board-game interventions to support early mathematical reasoning.

Consistent with prior research, board games in early childhood have been shown to foster mathematical thinking. A systematic review of 19 studies by Balladares et al. (2024) reported significant gains in early mathematics learning, while Ramani and Siegler (2008, 2011) demonstrated improvements in number-line estimation, numeral identification, and early arithmetic through linear number board games. Together, these findings highlight the role of games involving numbers, shapes, and spatial relations in supporting foundational mathematical skills.

The results further indicated that intervention effects varied according to children’s initial performance levels. Multiple regression and Johnson-Neyman analyses revealed that children with medium to high baseline skills benefited more from the intervention, underscoring the importance of developmental readiness. This pattern is consistent with research showing that play-based interventions are more effective once children reach certain cognitive thresholds, particularly in areas such as problem-solving and strategic thinking (Alotaibi, 2024; Ramani and Siegler, 2008). Moreover, the findings align with evidence identifying initial cognitive abilities as strong predictors of later academic achievement (Clements and Sarama, 2020; Nguyen et al., 2016). Finally, the study ex-tends the literature by addressing not only whether the intervention was effective, but also for whom.

The subdimension results showed that the intervention was more effective in data analysis-probability than in measurement, likely due to differences in game characteristics, developmental readiness, and domain-specific cognitive demands. Data analysis and probability skills (e.g., pattern recognition, classification, prediction) emerge naturally through gameplay. Research indicates that probability understanding in children under six is intuitive and variable (Nikiforidou and Pange, 2010; Way, 2003), and that five-year-olds can demonstrate basic probabilistic reasoning, contrary to Piagetian views (Nikiforidou and Pange, 2009). Consistently, Ergül and Artan (2015) found that 60-74-month-old children’s responses varied with the type and amount of probabilistic information.

In the present study, several board games (e.g., Q-bitz Junior, Rondo Vario, Man-gala) required children to identify patterns, anticipate outcomes, and make strategic decisions, thereby supporting skills related to ordering, grouping, and prediction. These competencies closely align with the data analysis-probability subdimension, including shape classification, graph interpretation, and probabilistic reasoning. Prior research similarly highlights that such experiences provide a strong foundation for early probability and data interpretation skills (Ramani and Siegler, 2008, 2011). Moreover, the data analysis-probability tasks in the assessment tool (Ergül and Artan, 2015) parallel the cognitive processes frequently engaged during gameplay, making the relatively strong intervention effects in this subdimension theoretically plausible.

The limited improvement in the measurement subdimension is consistent with Balladares et al.’s (2024) systematic review, which noted that board-game-based interventions do not affect all mathematical subdomains equally. Measurement concepts (e.g., length, weight, area, volume, time) rely on concrete physical experiences, com-parison, and the use of measurement tools, whereas board games tend to involve more abstract or symbolic activities. As a result, opportunities to experience measurement concepts directly within gameplay are limited. Similar findings have been reported in prior studies, suggesting that while board games support numerical reasoning, spatial cognition, and strategic thinking (Clements and Sarama, 2020), additional experiences are often required for measurement learning (Platas, 2018). Supporting this interpretation, studies using the same assessment tool (Ergül and Artan, 2015) found that children particularly struggled with time-related items, likely due to developmental limitations in understanding abstract time concepts.

Moreover, as practitioners frequently emphasize, children need opportunities for physical, hands-on experiences in order to consolidate measurement concepts (Platas, 2018). Given that the intervention provided limited opportunities for explicit measurement tasks (e.g., timing, length comparison), the more modest gains in measurement skills are expected. As noted by Ergül and Artan (2015), concepts such as time are inherently difficult for young children and are less frequently reinforced through play. Thus, the diminished impact on the measurement subdimension in this study likely reflects both the content of the selected games and children’s developmental progression.

## 5 Limitation and suggestions

This study includes several limitations that should be considered when interpreting the findings. First, the relatively small sample size reflects the pilot and exploratory nature of the study and limits the generalizability of the findings. Nevertheless, the study provides important preliminary evidence and effect size estimates that may inform future large-scale experimental research. In addition, participants were nested within intact classrooms, for which multilevel analyses are typically recommended. However, because only two classrooms were included, multilevel modeling was not feasible. Consequently, classroom-level effects could not be explicitly modeled, and interaction effects were examined using the Johnson-Neyman technique within a single-level analytic framework.

Another limitation is that only short-term effects were assessed; therefore, the long-term sustainability of gains in mathematical reasoning remains unclear. Longitudinal studies are needed to examine whether these effects persist over time. Furthermore, measurements were conducted within a specific age group and cultural context, which may limit external validity. Since play experiences can vary across cultures, replication studies in different settings are warranted. Sampling from a single school also restricts the transferability of the findings to broader populations.

The use of intact classrooms without random assignment constitutes an additional limitation. Although baseline equivalence between groups was established through pretest comparisons and standardized procedures were applied throughout the intervention, potential classroom or teacher effects cannot be fully ruled out. The study also relied on a single measurement tool and lacked qualitative data, thereby limiting the depth of interpretation. Future research could examine the effectiveness of pre-intervention models designed for children with low initial levels and investigate moderators (initial level, working-memory capacity, prior experience) and mechanisms (e.g., strategy acquisition, metacognitive awareness) in greater detail.

Finally, the intervention showed limited impact for children with low initial performance levels. Future research should investigate pre-intervention support models for children with lower baseline skills and examine moderators such as working-memory capacity, prior experience, and strategy use in greater detail. In addition, although the games were particularly effective for data analysis–probability skills, they were less effective for measurement skills, which require more concrete experiences. Early childhood educators should integrate physical materials (e.g., rulers, cups, balance scales) and measurement-focused play (e.g., length or volume comparison, sand-timer activities) to promote more balanced mathematical reasoning gains.

## Data Availability

The data analyzed in this study is subject to the following licenses/restrictions: the data that support the findings of this study are available on request from the corresponding author. Requests to access these datasets should be directed to SK, skarabekmez@gmail.com.
